# Hospital Festivals and Meetings

**Published:** 1895-01-19

**Authors:** 


### HOSPITAL FESTIVALS AND MEETINGS.

### MIDDLESEX HOSPITAL.—PRESENTATION TO LORD SANDHURST.

On Saturday last, at the Middlesex Hospital, an address and a handsome gift of plate, consisting of a silver bowl and four goblets, was presented to Lord Sandhurst on the occasion of his retirement from the chairmanship of the weekly board, owing to his appointment as Governor of Bombay.

Some thirty members of the weekly board, the medical and surgical staff, and the hospital staff were present in the board-room, including Mr. J. Bell Sedgwick (the deputy chairman), Colonel Needham, Sir Ralph Thompson, K.C.B., Mr. C. Kegan Paul, Mr. F. Clare Melhardo (secretary, superintendent), Miss Thorold (lady superintendent), and the Rev. W. G. Deighton.

Letters of regret were received from Mr. Francis Hoare, Mr. Fowler, and others, and the following address was then read by Mr. Melhardo:—

"To the Right Hon. Lord Sandhurst.

"We, the undersigned members of the weekly board, the honorary medical and surgical staff, and the officers of the Middlesex Hospital, congratulate your Lordship most heartily on the distinguished honour shown you by Her Majesty in your appointment as Governor of Bombay, although our congratulations are tinged with sincere regret at the loss we sustain by your consequent withdrawal from the chairmanship of our board. It is impossible to overstate our sense of the ability, kindness, courtesy, and tact, which you have shown in that office, and of the manner in which, while introducing many salutary reforms, you have broken no old traditions, and have aided in every way the harmonious working of the institution. We ask you to be so good as to accept the bowl and goblets which we offer you, and trust that they will serve occasionally to remind you of your colleagues whose sympathy and interest will never fail to accompany your career. We feel confident that in great matters of state you will show those same qualities which have gained you here our esteem and affection. (Signed) John Bell Sedgwick, Francis Hoare, G. J. Marjoribanks, Walter Rothschild, William Debenham, W. F. Larkins, Spencer Lyttelton, James G. Noel, Charles Hollway, William Hales (Master of the Drapers' Company), Griffith Jarrett, W. H. Burroughes, W. T. Houldsworth, F. Davis, S. B. Bancroft, Charles Cecil Cotes, Gathorne Hardy, Elliott Lees, Herbert Eaton, C. Kegan Paul, George B. Hudson, Charles Needham, W. E. Gillett, Ralph Thompson, J. H. Acheson, William C. Houghton, J. W. Hulke, George Lawson, William Cayley, Henry Morris, Andrew Clark, Sydney Coupland, R. Douglas Powell, J. Kingston Fowler, Cecil Y. Biss, William Lang, A. Pearce Gould, William Duncan, J. J. Pringle, W. Pasteur, Robert Boxall, Walter Essex Wynter, G. M. Thorold, E. A. Fardon, W. G. Deighton, F. Clare Melhardo."

Mr. Bell Sedgwick and Mr. J. W. Hulke both bore cordial testimony to the invaluable nature of the services rendered by Lord Sandhurst to the hospital.

Lord Sandhurst in reply said the occasion was to him a most affecting one, and he felt it deeply. For six years they had worked together most harmoniously and usefully in the public interest. With regard to the beautiful gifts which were before him, he should care for them not so much for their intrinsic beauty, but as a sign that his work there was estimated at some considerable value. It had always been his proud boast that hospital managers got nothing whatever out of their work, but this gift had been prompted by feelings of simplicity and generosity, and he appreciated it accordingly. He proposed to have the names of the givers engraved on the plate, and so far as it rested with him, it should remain always in his family. Referring to his past work, Lord Sandhurst said he had succeeded Major Ross as chairman, by whom things had been made extremely easy for him. From that time until he accepted office under the Government he could truthfully say that the work occupied most of his thoughts. There was no one connected with the hospitnl from whom he had not received the greatest courtesy at all times. Differences of opinion must needs occur, but "difficulties were made to be got over," and in any they had had he had never been afraid to hear those who had views of their own, and he could not call to mind any one occasion when such a difference of opinion had left any feeling behind. With regard to the administrative staff, the chairman was naturally more frequently in the hospital and in communication with the secretary-superintendent and the lady superintendent than other members of the board, and he could say that it was impossible to have any hospital more disinterestedly and loyally served by its officers than was the Middlesex Hospital. The public had great confidence in it and in its management as was shown by increased funds. He had little more to say, except that he left the chair which he now occupied for the last time, with very great regret. The six years during which he had been chairman had entailed some work, but also an enormous amount of pleasure and interesting experience. He left the hospital with deep personal regret and the sincerest feeling of good-will to all, and in now saying farewell he did so individually and collectively.

The inscription upon the silver bowl is as follows: "To the Right Hon. Lord Sandhurst. This bowl and the accompanying four goblets are presented with the sincere esteem and regard of his colleagues at the Middlesex Hospital. 12th January, 1895."

